# Identification of candidate single-nucleotide polymorphisms (SNPs) and genes associated with sugarcane leaf scald disease

**DOI:** 10.1038/s41598-024-67059-w

**Published:** 2024-07-13

**Authors:** Yisha Li, Pingping Lin, Qian You, Jiangfeng Huang, Wei Yao, Jianping Wang, Muqing Zhang

**Affiliations:** 1 Guangxi Key Laboratory for Sugarcane Biology, College of Agriculture, Guangxi University, Nanning, 530005 China; 2 Agronomy Department, IFAS, University of Florida, Gainesville, FL 32611 USA

**Keywords:** Plant breeding, Plant genetics

## Abstract

Leaf scald, caused by *Xanthomonas albilineans*, is a severe disease affecting sugarcane worldwide. One of the most practical ways to control it is by developing resistant sugarcane cultivars. It is essential to identify genes associated with the response to leaf scald. A panel of 170 sugarcane genotypes was evaluated for resistance to leaf scald in field conditions for 2 years, followed by a 1-year greenhouse experiment. The phenotypic evaluation data showed a wide continuous distribution, with heritability values ranging from 0.58 to 0.84. Thirteen single nucleotide polymorphisms (SNPs) were identified, significantly associated with leaf scald resistance. Among these, eight were stable across multiple environments and association models. The candidate genes identified and validated based on RNA-seq and qRT-PCR included two genes that encode NB-ARC leucine-rich repeat (LRR)-containing domain disease-resistance protein. These findings provide a basis for developing marker-assisted selection strategies in sugarcane breeding programs.

## Introduction

Sugarcane is an important commercial crop belonging to the *Poaceae* family. It is cultivated in tropical and subtropical regions worldwide^1^. Being a dual-purpose crop, it accounts for 85% of global sugar production and 40% of bioenergy feedstock for ethanol production^2^. As the third-largest global sugarcane producer, China contributes 5% of the total sugar production worldwide (FAO, http://www.fao.org/faostat). Despite its economic significance, sugarcane is susceptible to various pathogens, resulting in yield losses, sucrose reduction, and economic setbacks^3^. *Xanthomonas albilineans* is a xylem-intruding bacterium that causes sugarcane leaf scald, a severe disease in 66 countries^4^. When the pathogen colonizes the plant’s vascular system, it forms a white, narrow, and sharply defined stripe. This infection ultimately leads to complete wilting and necrosis in the diseased leaves, posing a severe threat to susceptible sugarcane genotypes^5^. A recent report of a leaf scald outbreak in southern Mexico indicated a 3 to 15% incidence of leaf scald in the infected fields^6^. Leaf scald was first reported in China in 2016 and has subsequently spread and escalated into an epidemic, resulting in a severe threat to sugar production^5,7,8^. The most cost-effective and environmentally friendly strategy to manage the permanent risk of leaf scalds is to develop resistant cultivars^9,10^. However, the troublesome aspect of resistance evaluation is that symptom expression is strongly affected by environmental conditions^11^. The unstable symptom expression results in an inability to detect susceptibility accurately; thus, multiple field trials are needed. Furthermore, utilizing molecular markers associated with resistance can significantly contribute to marker-assisted sugarcane breeding programs. Genetic markers, especially DNA markers, have become essential and efficient tools in developing disease resistance^12^ and are more conducive to developing new varieties with stable resistance than traditional breeding methods^13^.

Sugarcane has a complex and large genome (approximately 10 Gb) because sugarcane is derived from interspecific hybridization between the high-sugar species *Saccharum officinarum* and the wild species *S. spontaneum*, followed by several backcrosses with *S. officinarum*, making genetic and genomic studies of sugarcane cultivars challenging^14,15^. However, target enrichment sequencing is an ideal and cost-effective approach for sequencing hundreds of putatively single-copy nuclear regions in multiple samples^16^. SNP genotyping platforms are based on target enrichment sequencing, which is very flexible regarding the number of SNP markers. The Axiom sugarcane 100K SNP is an efficient genetic tool for high-throughput genotyping in highly polyploid sugarcane^17^. The Axiom sugarcane 100K SNP arrays were designed using a large number of single nucleotide polymorphisms (SNPs) markers discovered from sugarcane genomes using the sorghum (*Sorghum bicolor*) genome as reference^18,19^, which is a closely related crop containing 95.2% identical coding regions with sugarcane^20^. Most of the markers were developed using mapping populations derived from bi-parental crosses. In constructing a genetic linkage map, a 3:1 (resistant: susceptible) segregation was observed in the sugarcane cultivar “R570” for rust resistance, indicating the possible presence of dominant resistance genes^21^. Eight QTLs associated with leaf scald response were identified, explaining 5.23% to 16.93% of the phenotypic variance^22^. Eighteen QTLs regulated resistance to *sugarcane yellow leaf virus*, segregating the two mapping populations containing 27 disease-resistant genes^17^. These successful examples provide feasibility for marker-assisted selection in sugarcane. In addition, genome-wide association studies (GWAS) have also become a popular tool for identifying the genetic factors underlying marker-trait associations and specific genomic regions responsible for the essential genes associated with various crop diseases and complex traits ^23–25^.

To investigate candidate loci and genes related to resistance against leaf scald, we genotyped 170 sugarcane germplasms using an Axiom Sugarcane 100K SNP array. The genotypes were evaluated for leaf scald resistance in field conditions over 2 years (2021–2022), followed by one greenhouse experiment. We used five association models of GAPIT^26^ to identify SNP loci associated with leaf scald resistance. Building on this, we explored candidate resistance genes by integrating gene annotation information with the publicly available transcriptome. Our study describes promising results that will accelerate marker-assisted selection for improving leaf scald resistance in sugarcane breeding programs and delineate prospective targets for cloning novel resistant genes.

## Results

### Phenotypic evaluation for leaf scald

The disease incidence (IC) and index (DI) of leaf scald in each experiment have been listed in Supplementary Table 1. Analysis of variance for IC and DI indicated that the genotypes (G), environment (E), and G × E effects were highly significant (p < 0.001; Supplementary Table 2). The IC distribution of sugarcane genotypes ranged from 0.00 to 21.43%, and the DI distribution ranged from 0.00 to 12.00 (Table 1). IC and DI values were continuous in all environments, indicating that the traits were quantitative. The broad-sense heritability (*H*_*B*_^2^) for IC and DI over two consecutive years in the field were 0.58 and 0.70, respectively. Moreover, *H*_*B*_^2^ for IC and DI of the greenhouse experiment were 0.72 and 0.84, respectively, indicating that genetic factors played an important role (Table 1).

**Table 1:** Phenotypic variation two measures of leaf scald disease resistance in the 170 genotypes of the GWAS panel.

Trait	Experiment	Max	Min	Mean	Stdev	CV	*H*_*B*_^2^
Disease incidence	2021	21.43	0.00	3.49	4.14	1.19	0.58
	2022	16.00	0.00	3.16	2.85	0.90	
	Greenhouse (GH)	20.00	0.00	4.05	3.63	0.90	0.72
Disease index	2021	12.00	0.00	1.59	2.00	1.26	0.70
	2022	10.66	0.00	1.41	1.44	1.02	
	Greenhouse (GH)	12.00	0.00	1.72	1.76	1.03	0.84

Although the correlation between IC and DI of the cultivars was significant between field and greenhouse studies, the actual differences in the average IC and DI among genotypes were much more minor in the field trial than in the greenhouse trial (Supplementary Table 1). The phenotyping results of most genotypes were similar. Thirty-two genotypes were symptomless or immune in all three environments. The genotype CP97-1602 was the most severe in all three environments. Specific genotypes showed inconsistent phenotypes across the three environments. For instance, seven genotypes (4–91, 4–195, 5–77, 4–117, GT07-713, GT02-1156, 2–65) were asymptomatic in a field environment (2022), but showed typical symptoms in greenhouse environments (Supplementary Table 1).

**Figure 1:**
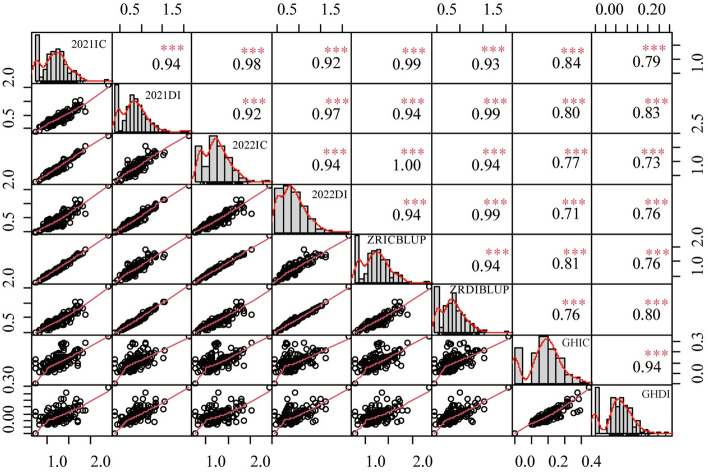
Genotypic correlation between disease severity evaluated in different experiments. The diagonal panels show histograms for each environment trait. The lower and upper triangular panels show scatter plots and Pearson correlation coefficients between pairs of environment traits, respectively. ***Indicates significance at 0.001 levels. The X and Y-axis represent the numerical values.

The distribution of the raw phenotypic data for leaf scald was visualized using the histogram, which showed continuous distribution variation and left skewed in each experiment of 170 genotypes (Supplementary Fig. 1). To achieve normality of residuals, BLUP values were calculated by applying a mixed linear model, and the data from the greenhouse experiment were transformed using an inverse sine square root transformation. Ultimately, eight data sets were used for subsequent GWAS analysis, including the value of genotype by year interaction, BLUP, and transformed phenotypic data from the greenhouse (Fig. 1, Supplementary Table 3). Correlations between IC and DI within transformed data ranged from 0.73 to 0.99. Pearson’s correlation coefficient among different datasets indicated a highly significant (p < 0.001) correlation (Fig. 1).

### Population structure

**Figure 2:**
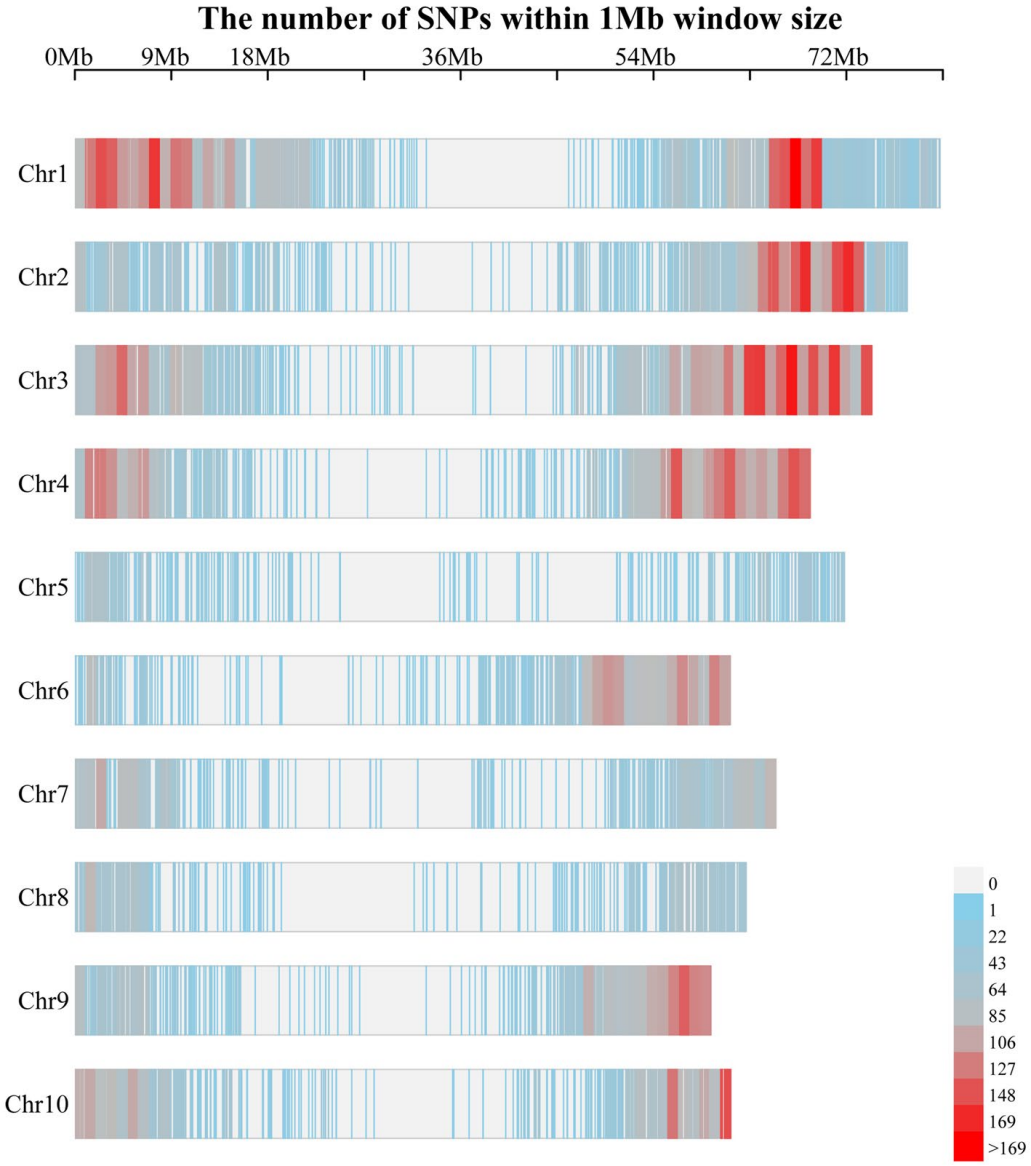
Distribution of high-quality SNPs over ten sorghum chromosomes.

**Figure 3:**
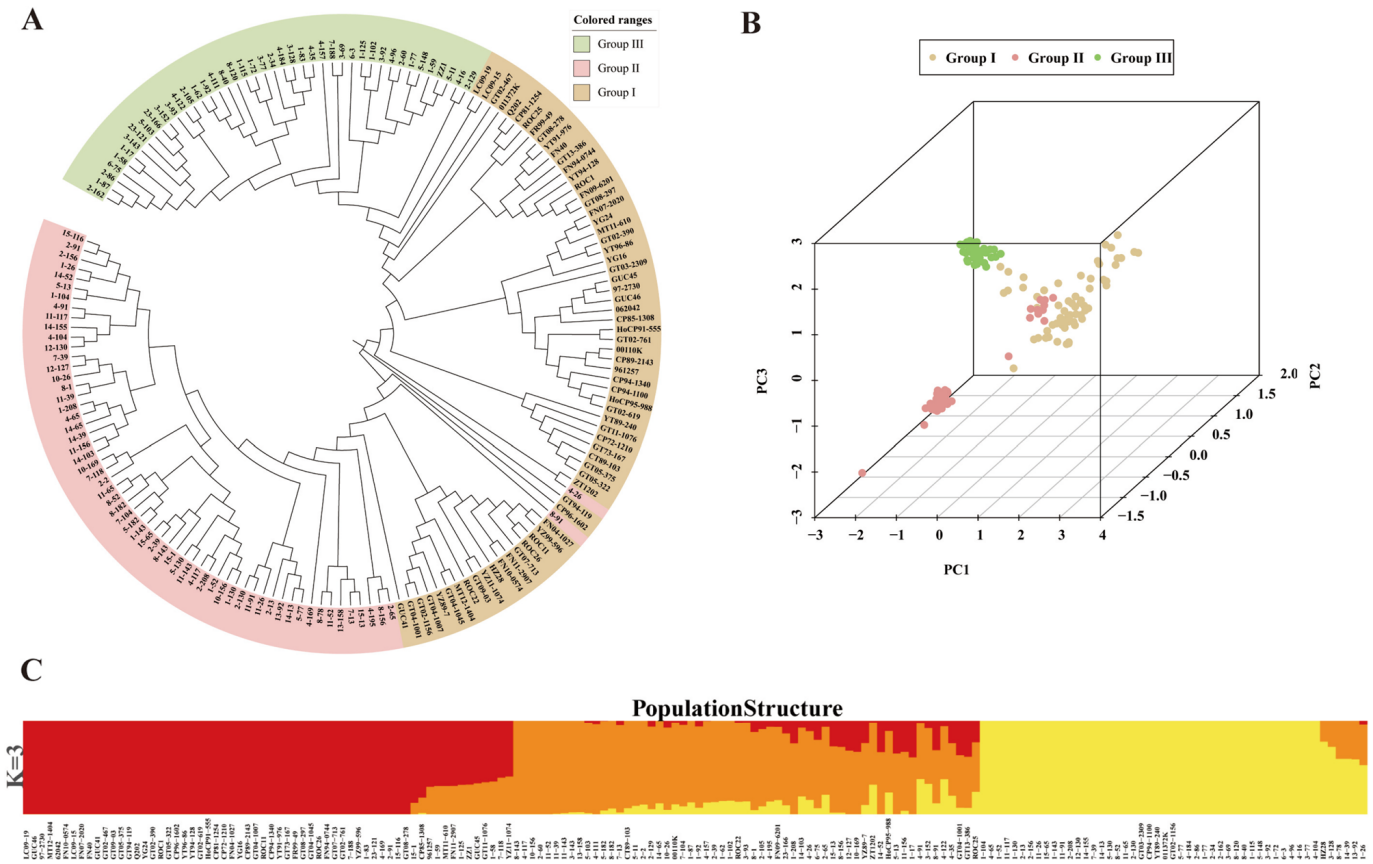
Phylogenetic and population structure analysis of 170 sugarcane genotypes based on 26,787 SNP markers. (**A**) Phylogenetic tree of the 170 genotypes; (**B**) scatterplot based on principal component analysis; (**C**) stacked bar plots of ADMIXTURE results for K = 3.

A total of 100,097 SNPs were filtered to generate 26,787 high-quality SNPs for subsequent analysis. These SNPs were distributed across ten sorghum chromosomes, with the highest number of SNPs on chromosome 1 (Fig. 2). The pairwise kinship matrix heatmap of the 170 genotypes revealed three significant subgroups, with their relationships displayed along the diagonal line with a few large blocks of closely related individuals (Supplementary Fig. 2). Moreover, cross-validation (CV) error was used to predict the most appropriate value for K (the optimal number of populations in the dataset). According to admixture clustering, a break in the slope was observed at K = 3, showing the lowest CV error (Supplementary Fig. 3). For the phylogenetic tree, lines with different colors indicated different subgroups, where genotypes 4–26 and 8–91 of Group II were clustered into Group I (Fig. 3A). Principal component analysis (PCA) plot for all genotypes clearly showed that samples from the same subgroups clustered together (Fig. 3B). The admixture plots showed results for K = 3, the lowest cross-validation (CV) error (Fig. 3C), revealing a high degree of admixture in many genotypes, with 23 (35%) genotypes in group I, 57 (93.44%) genotypes in group II, and 27 (62.8%) genotypes in group III, respectively, mixed with another group. In particular, most genotypes in groups II and III were mixed. This result may be due to these genotypes inherited from a common ancestry. According to the clustering, kinship analysis, phylogenetic tree construction, and PCA analyses, parental origin was the primary factor determining this panel’s classification diversity.

### Genome-wide association analysis

**Figure 4:**
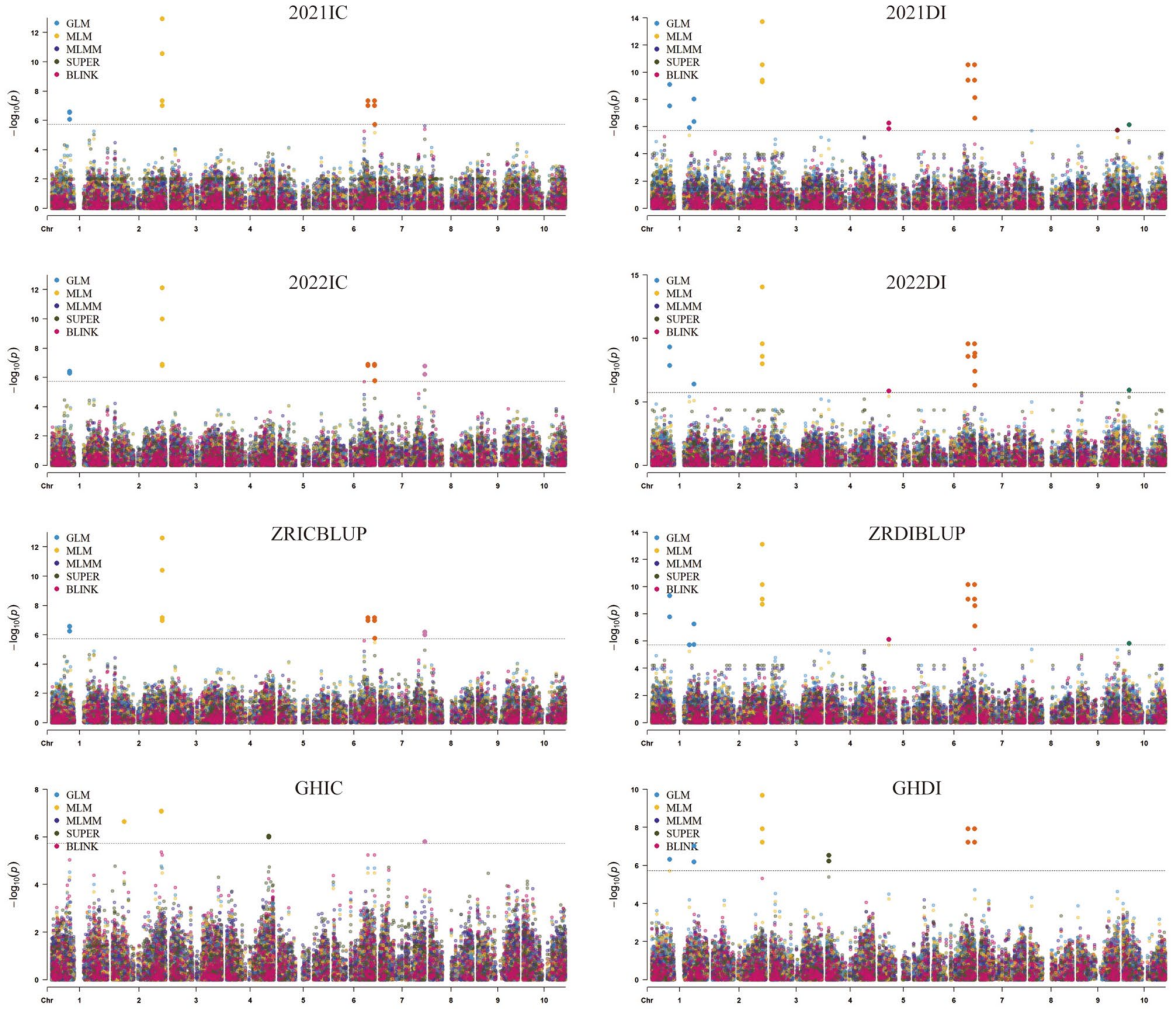
Manhattan plots of GWAS results showing significant SNPs associated with leaf scald resistance in sugarcane genotypes diversity panel. The X-axis shows the distribution of SNPs across 10 chromosomes, while the Y-axis shows the Bonferroni corrections threshold. The black dashed horizontal line depicts the uniform significance threshold (− Log10 p-value = 5.73).

Five GWAS models were tested, and QQ plots for each trait were compared to assess the optimal model for controlling false-positive associations due to the structure and relatedness (Supplementary Fig. 4). A total of 107 SNPs were identified, of which 41 were by GLM, 35 by MLM, 12 by MLMM, 7 by SUPER, and 12 by BLINK (Table 2, Supplementary Table 4). After analysis, most SNPs were overlapping; thirteen significant SNPs were associated with the response to leaf scald (Table 3, Fig. 4). Eight SNPs were detected in multi-environments and -association models, including S1_25897077, S1_61002332, S2_72384593, S5_14567373, S6_50836650, S6_60340985, S6_60594020, S7_65315409. The other five SNPs were detected in a single environment or association model. The thirteen candidate SNPs were located on seven sorghum chromosomes (Chr01, Chr02, Chr04, Chr05, Chr06, Chr07, Chr10). Most were located on Chr02 and Chr06, each containing three SNPs (Table 3). The phenotypic variation explained (PVE) by a single SNP ranged from 4.28 to 17.98%; eight SNPs had a PVE greater than 10% (Table 3). These significant SNPs and their − log10 p-values were visualized in the Manhattan plots (Fig. 4).

**Table 2:** Statistics of genome-wide association analysis results from five different methods.

Trait	Experiment	GLM	MLM	MLMM	SUPER	BLINK	Total	
Disease incidence	2021	4	4	1	2	1	12	46
	2022	5	4	2	1	2	14	
	ZRICBLUP	5	4	2	1	2	14	
	Greenhouse (GH)	0	0	3	3	0	6	
Disease index	2021	7	7	1	0	2	17	61
	2022	7	5	1	0	3	16	
	ZRDIBLUP	7	6	1	0	2	16	
	Greenhouse (GH)	6	5	1	0	0	12	
Total		41	35	12	7	12	107

**Table 3:** SNPs significantly associated with leaf scald resistance identified by GWAS analysis.

SNP marker	Chr	Pos	MAF	P-value	PVE (%)	Experiment-traits	Association model
S1_25897077	1	25,897,077	0.49	7.81E−15	8.55	2021IC, 2021DI, 2022IC, 2022DI, GHDI, ZRICBLUP, ZRDIBLUP	GLM, MLM, SUPER
S1_61002332	1	61,002,332	0.49	2.55E−09	9.50	2021DI, 2022DI, GHDI, ZRDIBLUP	GLM, MLM
S2_72384593	2	72,384,593	0.50	7.59E−14	14.54	2021IC, 2021DI, 2022DI, GHDI, ZRICBLUP, ZRDIBLUP	GLM, MLM, BLINK, MLMM
S5_14567373	5	14,567,373	0.49	5.50E−08	4.28	2021DI, 2022DI, ZRDIBLUP	GLM, BLINK
S6_50836650	6	50,836,650	0.50	6.83E−11	17.89	2021IC, 2021DI, 2022IC, 2022DI, GHDI, ZRICBLUP, ZRDIBLUP	GLM, MLM
S6_60340985	6	60,340,985	0.50	6.83E−11	16.37	2021IC, 2021DI, 2022IC, 2022DI, GHDI, ZRICBLUP, ZRDIBLUP	GLM, MLM
S6_60594020	6	60,594,020	0.49	1.27E−09	11.04	2021IC, 2021DI, 2022IC, 2022DI, ZRICBLUP, ZRDIBLUP	GLM, MLM, BLINK, SUPER
S7_65315409	7	65,315,409	0.34	5.39E−07	11.50	2021DI, 2022IC, GHIC, ZRICBLUP	SUPER, BLINK, MLMM
S2_71293304	2	71,293,304	0.23	8.16E−08	11.79	GHIC	SUPER, MLMM
S4_3084020	4	3,084,020	0.49	2.88E−07	14.36	GHDI	GLM, MLM
S4_61259456	4	61,259,456	0.34	9.31E−07	12.72	GHIC	SUPER, MLMM
S10_8458899	10	8,458,899	0.49	1.48E−06	6.12	2021DI, 2022DI, ZRDIBLUP	BLINK
S2_17206572	2	17,206,572	0.15	2.24E−07	17.98	GHIC	MLMM

*Chr* chromosomes, *Pos* position, *MAF* minor allele frequency, *PVE* Phenotype_Variance_Explained.

### Candidate gene for leaf scald resistance

**Figure 5:**
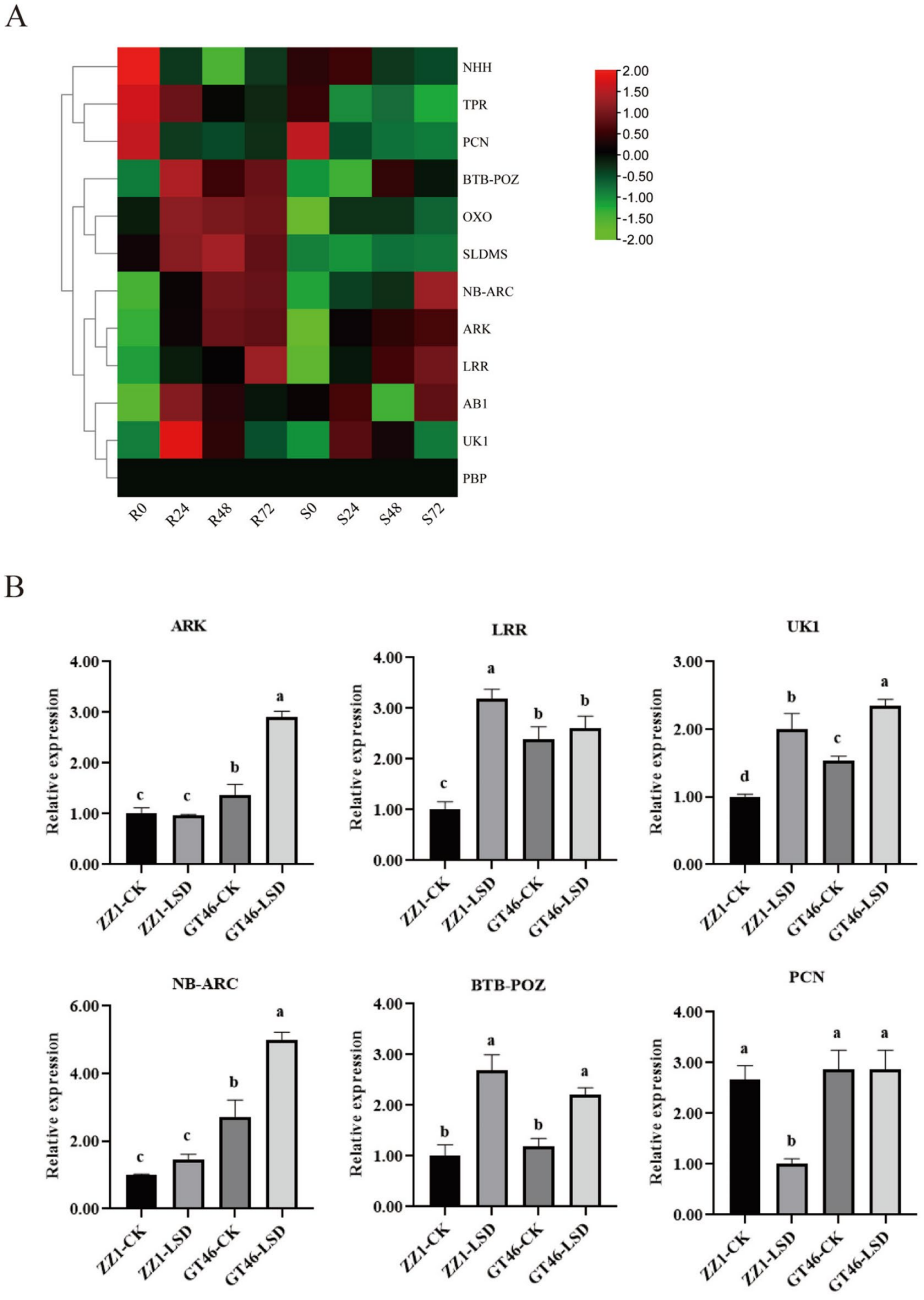
The expression pattern of the candidate genes observed in (**A**) transcriptome and (**B**) quantitative reverse transcription-PCR (qRT-PCR). Each column of (**A**) represents a time point after bacterial inoculation in one cultivar; S-susceptible cultivar (ROC20); R-resistant cultivar (LCP 85–384); 0, 24, 48, and 72 indicates hours post-inoculation. For (**B**), the abscissa represents cultivar (ZZ1, GT46) and treatment (CK, un-inoculated; LSD, inoculated), and the ordinate represents the relative expression of genes calculated by the 2 − ^ΔΔCT^ method. Different letters (a, b, and c) represent statistically significant differences (ANOVA with Turkey post-hoc analysis, 5% level). The mean ± S.D. of the biological replicates is presented.

Of the thirteen significant SNPs, only one SNP (S10_8458899) was identified in the intergenic spacer region. The remaining 12 significant SNP markers were located within genes and were considered candidate genes (Table 4). SNP analysis revealed diverse variant classifications, including four missense variants, two three-prime UTR variants, three intron variants, and three synonymous variants. The SNP with the largest log10 (p) value found in this study was marker S1_25897077, situated within the gene BTB-POZ (gene symbol) sequence, predicted to code a BTB-POZ and MATH domain. In addition, SNP markers S1_61002332 and S2_71293304 were found within genes coding for an Oxoprolinase (OXO) and Tetratricopeptide repeat (TPR)-like superfamily protein (TPR), respectively. Two SNPs (S5_14567373 and S6_50836650) were identified inside the gene encoding for putative proteins with LRR and NB-ARC domains, known for their essential role in disease resistance. Finally, SNP marker S7_65315409 was located within gene SLDMS (gene symbol), predicted to code for an S-adenosyl-l-methionine-dependent methyltransferase superfamily protein. Moreover, the CDS sequences of the sorghum genome were BLASTed to the *S. spontaneum* and *S. officinarum* genome separately. Twelve sorghum candidate genes exhibited 30 and 102 homologous genes in *S. spontaneum and S. officinarum,* respectively (Supplementary Table 5). We conducted a detailed analysis of candidate genes, integrating GWAS-identified genes with differentially expressed genes (DEGs) from RNA-seq. Among the twelve candidate genes, the six genes (NHH, TPR, PCN, BTB-POZ, OXO, SLDMS) were found to show relatively higher expression levels in resistant cultivar (LCP 85–384), gene PBP showed a shallow level or no expression in both susceptible cultivar (ROC20) and resistant cultivar (LCP 85–384) (Fig. 5A). In addition, we analyzed the differential expression of twelve genes in both cultivars at different inoculation time points, and the results show that five genes displayed significant differential expressions at least at a one-time point in both susceptible cultivar (ROC20) and resistant cultivar (LCP 85–384) (Supplementary Table 6).

**Table 4:** The positions of SNP markers and sorghum genes overlapping, potentially associated with leaf scald resistance.

SNP Marker	Alleles	Variant classification	Linked Sorghum genes and position/model				Gene symbol	Gene description
S1_25897077	T/C	Missense variant	Sobic.001G246600	25,896,406	25,897,510	+	BTB-POZ	BTB-POZ and MATH domain 2
S1_61002332	G/A	Synonymous variant	Sobic.001G322700	60,999,232	61,003,989	+	OXO	Oxoprolinase 1
S2_17206572	G/A	3 Prime UTR variant	Sobic.002G126300	17,206,479	17,211,433	−	UK1	Unknown
S2_71293304	A/C	Intron variant	Sobic.002G347600	71,290,012	71,298,531	−	TPR	Tetratricopeptide repeat (TPR)-like superfamily protein
S2_72384593	T/C	Intron variant	Sobic.002G362800	72,382,734	72,395,027	−	AB1	Actin binding
S4_3084020	T/C	Intron variant	Sobic.004G037900	3,080,556	3,090,663	−	ARK	Arabinose kinase
S4_61259456	A/G	Synonymous variant	Sobic.004G268400	61,243,436	61,261,443	+	PCN	P-loop containing nucleoside triphosphate hydrolases superfamily protein
S5_14567373	A/C	Missense variant	Sobic.005G094001	14,565,253	14,571,702	−	LRR	LRR and NB-ARC domains-containing disease resistance protein
S6_50836650	A/G	3 Prime UTR variant	Sobic.006G146700	50,836,398	50,840,606	−	NB-ARC	NB-ARC domain-containing disease resistance protein
S6_60340985	C/T	Missense variant	Sobic.006G271600	60,337,825	60,343,863	−	PBP	Poly(A) binding protein 2
S6_60594020	C/A	Missense variant	Sobic.006G274800	60,591,957	60,597,186	+	NHH	Nudix hydrolase homolog 26
S7_65315409	G/A	Synonymous variant	Sobic.007G225800	65,311,844	65,318,939	+	SLDMS	S-Adenosyl-l-methionine-dependent methyltransferases superfamily protein

− represent minus strand, + represent plus strand.

### Validation of candidate gene expression

A complete list of Ct values, including reference genes and candidate genes, is given in Supplementary Table 8. The quality of primers was tested by examining the formation of primer dimers based on the dissolution curves. All primer pairs with a single high peak demonstrated no nonspecific amplification (Supplementary Fig. 5). Six candidate genes were selected for qRT-PCR validation. Under normal conditions without *Xanthomonas albilineans* stress, there are four genes (ARK, LRR, UK1, and NB-ARC) showed significant differences in the relative expression levels of the resistant (ZZ1) and susceptible (GT46) genotypes. In contrast, the genes BTB-POZ and PCN showed no significant difference in the relative expression levels. The genotypes ZZ1 did not show a significant difference in the relative gene expression level of genes ARK and NB-ARC compared with control when subjected to inoculation treatment. However, there was a difference in the relative expression levels in GT46 compared with control when subjected to inoculation treatment of these genes. One of the genes coded LRR domains and gene BTB-POZ were upregulated in resistant genotype ZZ1. Another gene, NB-ARC, was expressed significantly higher in the susceptible genotype GT46 than in the resistant genotype ZZ1. The gene PCN encoded a P-loop containing superfamily protein of triphosphate hydrolases, which was significantly downregulated in resistant genotype ZZ1. In addition, the genes involved in coded LRR and PCN were not differentially expressed after inoculation in GT46 (Fig. 5B). This further proves that the six putative genes were closely associated with the response to leaf scald.

## Discussion

Severe yield losses in China have been reported due to leaf scald disease^5,8^. Consequently, future outbreaks of leaf scalds represent a persistent danger to yield and quality stability. It is urgent to breed resistant varieties due to the rapid evolution of virulent races of the pathogen. Genome-wide association mapping (GWAS) is a powerful approach that facilitates identifying genes regulating several traits. To develop the genetic markers linked to the leaf scald resistance for marker-assisted selection in the breeding programs, we used the genetic data available for 170 genotypes tested for leaf scald resistance in three different environments. Our results could help understand the resistance mechanism of leaf scald and accelerate the sugarcane breeding process toward combating this disease.

A high phenotypic variation was observed within the 170 genotypes. We calculated BLUP values through two consecutive years in the field to reduce the environmental impact. The frequency distribution for BLUP values followed a biased normal distribution, similar to the previous studies^27,28^. Despite significant genotype-by-environment interaction, the correlation of leaf scald severity across different experiments was high, indicating the significance of the evaluation method for leaf scald. The high heritability highlighted the importance of genetic factors and the reliability of the phenotypes. These findings collectively suggested that the observed phenotypic variation could be attributed to the differences at the genotypic level.

SNP microarray-based genotyping is widely used in crop genetic studies, including genome-wide association analysis (GWAS), linkage disequilibrium map, genome selection, and population structure^18,29,30^. Due to the lack of a high-quality sugarcane genome as a reference in earlier studies, the researchers developed an Axiom Sugarcane 100K SNP microarray using sorghum as a reference genome, which was applied to QTL localization and GWAS analysis of sugarcane traits^17^. The LD caused by population structure could promote false positive detection in GWAS analysis^31,32^. To avoid these false associations, the models consider covariates (population structure matrix and/or kinship matrix) to adjust the association tests on markers. When analyzing the population structure of genotypes, we found that subgroups were directly related to their parental origin, which is consistent with other findings^33,34^.

Thirteen SNP markers associated with the response to leaf scald were observed, with eight SNPs stably detected across multi-environments and -association models. These SNPs linked to resistance against leaf scald hold potential applications in sugarcane breeding programs for marker-assisted selection. In addition, these SNPs could be used to develop a Kompetitive allele-specific PCR (KASP) assay, a technique well evidenced in wheat^35–37^. GWAS analysis identified SNPs in deciphering disease mechanisms and the SNPs within the gene, especially the genes associated with the LD regions that might be involved in plant disease resistance^31,32^. However, current studies on LD decay distances in sugarcane have shown distances of 5 kb, 10 kb, 50 kb, and even 3.5 Mb ^38–41^. In this investigation, only the gene containing significant SNP was regarded as candidate genes due to the inaccuracy of determining the LD decay distance owing to the small number of SNPs. Moreover, we aligned the coding sequence (CDS) of each candidate gene with the *S. spontaneum* genome (AP85–441) and the *S. officinarum* genome, and the result showed that sorghum candidate genes had homologous genes in both *S. spontaneum* and *S. officinarum.* Therefore, these consistent corresponding homologous genes indicated that the SNP array hybridization results were accurate and reliable to a certain extent.

Genes identified by GWAS did not explain the gene’s contribution to the phenotype, necessitating a combination of gene expression patterns^42^. In this study, the expression levels of the candidate genes were analyzed using publicly available leaf scald disease-related transcriptomes. Six overlapping genes were generated after combining the GWAS-identified and differentially expressed genes (DEGs) of RNA-seq. Furthermore, differentially expressed candidate genes in resistant and susceptible varieties provide ideal molecular markers for identifying loci associated with leaf scald resistance in breeding. Therefore, all six genes were verified for resistance response to leaf scald in resistant and susceptible genotypes using qRT-PCR experiments. After functional annotation and categorization of candidate genes, we found that many genes are directly or indirectly associated with plant disease resistance. In a previous study, the broad-spectrum resistance genes contained a nucleotide-binding site and leucine-rich repeat domains- similar to the ones identified in this study^43^. Activated NB-LRRs represent a tip of the signaling cascade that triggers defense responses and not the causal genes defining the resistance alone^44,45^. Pathogens often have different infection strategies, while plants regulate their defense responses based on the nature of the pathogen and its pathogenic mechanisms. The genes PCN identified in the present study might play a negative regulatory role in leaf scald resistance. Moreover, the GWAS results will be used to analyze further resistant genes’ functions and regulatory mechanisms against leaf scald.

In conclusion, this study addressed the critical challenge of identifying resistant genes against leaf scald in sugarcane. Through evaluating a diverse panel of 170 sugarcane genotypes, thirteen significant SNPs associated with the response to leaf scald were discovered, with eight demonstrating stability across various environments and models. Combined with the data from the GWAS, transcriptome, and qRT-PCR, six candidate genes involved in the response to *Xanthomonas albilineans*, including two genes encoding NB-ARC leucine-rich repeat (LRR)-containing domain disease-resistance protein. All candidate SNPs and genes will provide a solid basis for implementing marker-assisted selection strategies in sugarcane breeding programs, offering a promising strategy for effective disease control and variety improvement.

## Materials and methods

### Plant materials

A total of 170 genotypes (local and abroad) were collected and kept in the sugarcane breeding base of Guangxi University. These core germplasm, including breeding lines, landraces, and commercial hybrids, have been selected after years of continuous evaluation for various traits. These genotypes were originated from 10 origins, namely United States of America (9), Australia (1), Sichuan (1), Yunnan (3), Guangdong (6), Fujian (9), Guangxi (135), Hainan (1) and Taiwan (5). Given different parental origins, 170 genotypes were subdivided into three distinct subgroups. Group I consists of 66 genotypes of the diversity panel, Group II consists of 61 progeny genotypes from the cross of ROC22 × CT103, and Group III consists of 43 progeny genotypes from the cross of ROC25 × YZ89-7. The list of genotypes is provided in Supplementary Table 1.

### Field evaluation

Field trials were performed in Fusui, China (107° 31′ E, 22° 17′ N) in a randomized complete block design with three replicates. Each plot consisted of a single-row zone with a length of 5 m and a row spacing of 2 m. Every plot was planted in 80 buds evenly over 5 m and separated by 1 m for every genotype. The natural occurrences of leaf scald were recorded for two consecutive years (2021–2022). Disease assessments were performed in April of each year, using the previously published method^9^, and recorded until it stabilized.

### Greenhouse evaluation

Evaluation of the leaf scald response of sugarcane to *Xanthomonas albilineans* was performed under a controlled greenhouse environment of the College of Agriculture, Guangxi University. The greenhouse experiments were set up using completely randomized blocks with three replicates. Each plot was 2 m long, 0.8 m wide, and spaced 1 m apart, and 40 buds were planted in each plot. The artificial inoculation was performed (in 2021) to verify the resistance of each genotype using the potent virulent strain of JG43^46^. The bacterial suspension was calibrated to 0.3 at OD_600_ using sterile distilled water. The sugarcane stalks were chopped into single buds, soaked in the bacterial solution for 3 h, and planted in a greenhouse at 28 °C with a relative humidity of 80%. The disease level was investigated using the previously published method^9^, and diseased plants were counted continuously from when the sugarcane grew to the 5-leaf stage (approximately one month after planting) until the disease level stabilized.

### Phenotypic statistical analysis

The severity of the leaf scald was evaluated as disease incidence and index. The following formulas calculated two disease indices: (1) Disease incidence (IC) = (ND/T) × 100; (2) Disease index (DI) = (1 × FL + 2 × ML + 3 × CB + 4 × N + 5 × D)/(5 × T) × 100), where ND = Number of diseased stalks, FL = the number of stalks with one or two streak lines, ML = the number of stalks with more than two streak lines, CB = the number of stalks with leaf chlorosis or bleaching, N = the number of stalks with leaf necrosis, D = the number of dead stalks or stalks with abnormal side shoots, and T = the total number of stalks. Variance analysis (ANOVA) was performed using the R software “multicomp” package (v4.1.2; R Core Team 2021). Field data were analyzed using a mixed model ANOVA, while greenhouse data were analyzed using one-way ANOVA. A mixed linear model was used to calculate the best linear unbiased predictors (BLUPs).

The model used for data analysis was as follows:

$${\text{Y}}_{{{\text{ijk}}}} = \mu + {\text{G}}_{{\text{k}}} + {\text{E}}_{{\text{i}}} + {\text{R}}_{{{\text{j}}({\text{i}})}} + {\text{E}}_{{{\text{Gik}}}} + \varepsilon_{{{\text{ijk}}}} ,$$

where Y_ijk_ is the observation of the kth genotype in the ith year in the jth replicate, μ is the overall mean, G_k_ is the effect of the kth genotype, Ei is the effect of the ith year, R_j(i)_ is the effect of the jth replication nested on the ith year, E_Gik_ is the effect of the interaction between the ith year and kth genotype, and ε_ijk_ is the effect of experimental error.

The broad-sense heritability (*H*_*B*_^*2*^) was estimated according to the formula:

$$H^{2} = \, \sigma_{{\text{g}}}^{{2}} /(\sigma_{{\text{g}}}^{{2}} + \sigma_{{{\text{ge}}}}^{{2}} /{\text{ne}} + \sigma_{{\text{e}}}^{{2}} /{\text{ne}} \times {\text{nr}}),$$

where *σ*_*g*_ genetic variance, *σ*_*ge*_ genetic and year interaction variance, *σ*_*e*_ error variance, *ne* the number of years, and *nr* the number of replicates.

All statistical models were fitted using the software package “asreml” within the R computing environment^47^. The heritability of the greenhouse experiment was calculated using the formula mentioned above. The phenotype data of the greenhouse experiment were directly transformed by arcsine square root to satisfy the model assumption of a normal distribution and input for the subsequent GWAS analyses. Correlation analysis of the different datasets was analyzed using the corrplot package in R software^48^.

### Genotyping and population structure analysis

The panel was genotyped with an Axiom Sugarcane 100K SNP array developed by Affymetrix using the Affymetrix GeneTitan® system. The physical positions of all the SNPs were based on sorghum genome v3.1.1. The Axiom Sugarcane100K SNP array obtained 100,097 SNPs. SNPs with a missing rate higher than 0.2 and a minor allele frequency (MAF) less than 0.05 were filtered out, resulting in 26,787 high-quality SNP markers for downstream analysis^17^. Based on the SNP markers obtained after filtering, neighbor-joining (NJ) tree construction was performed using MEGA-X software (model: Kimura 2-parameter; bootstrap: 1000) and edited with iTOL^49^. Principal Component Analysis (PCA) was performed using the R software and visualized and edited using the “ggplot2” R package. All genotypes were classified into three groups according to parental origin and labeled with different colors in the evolutionary tree and PCA graphs. Population structure analysis was performed by admixture software^50^, and the obtained results were visualized using the “ggplot” and “pophelper” R package^51,52^. The kinship matrix (K) was plotted using GAPIT's default parameters^26^.

### Genome-wide association analyses

Association analysis was performed using five association models of GAPIT, including the General Linear Model (GLM), Mixed Linear Model (MLM), Multiple-Locus Mixed Linear Model (MLMM), Settlement of MLM Under Progressively Exclusive Relationship (SUPER), and the Bayesian-information and Linkage-disequilibrium Iteratively Nested Keyway (BLINK) models^53–56^. The final trait values for the GWAS were derived from eight sets of data (Fig. 1, Supplementary Table 3). The first five PCA and Kinship results were the Q-Matrix and K-Matrix, respectively, as the variables were co-analyzed with genotype and phenotype combination. The results of GWAS were visualized using the “CMplot” R package (https://github.com/YinLiLin/R-CMplot). For GWAS results, a threshold of − log10 *p*-value ≥ 5.73 with Bonferroni correction (0.05/number of SNP markers) for each trait was considered significant for the association markers. The phenotypic variation explained by each SNP marker was calculated from the GAPIT^26^.

### Identification of candidate genes

Candidate genes were identified and described based on the general feature format (GFF) file from the sorghum genome v3.1.1, using TBtools software^57^. To assess the available candidate gene sequences from multiple species, we aligned the coding sequence (CDS) of each gene in the sorghum genome with that of its orthologous gene in the *S. spontaneum* reference genome (AP85–441) and *S. officinarum* (http://sugarcane.zhangjisenlab.cn/sgd/html/download.html). *S. spontaneum* and *S. officinarum* genes were screened with an e-value < 1e−06 and similarity ≥ 90% to the sorghum genes. The transcriptome data (PRJNA549590) was downloaded from the National Center for Biotechnology Information (NCBI) (https://www.ncbi.nlm.nih.gov/). The transcriptome consisted of leaf samples of resistant (LCP 85–384) and susceptible (ROC20) sugarcane cultivars infected by *Xanthomonas albilineans* collected at four-time points (0, 24, 48, and 72 h post-inoculation) with three biological replicates for transcriptome sequencing ^58^. All the qualified reads were aligned to the sorghum genome v3.1.1 as a reference genome using HISAT2 (https://ccb.jhu.edu/software/hisat2). Each leaf sample’s estimated transcript abundance (FPKM) was calculated using feature counts software with default parameters. Following the transcript abundance (FPKM), the calculated mean fold-change in expression values of differently treated samples of candidate genes were displayed in a heatmap using TBtools software ^57^. The candidate genes with p-value ≤ 0.05 and an absolute log2 fold-change ≥ 1.0 were considered to have statistically significant differential expressions.

### Validation of candidate genes by qRT-PCR

Based on the results of both GWAS and RNA-seq studies, six potential genes show significant differential expressions at least at one point time post-inoculation in both susceptible cultivar (ROC20) and resistant cultivar (LCP 85–384) and were chosen for qRT-PCR investigation. The CDS sequences of the candidate genes were extracted using TBtools software. Species-specific primer sets were designed using Vector NTI software; six pairs of specific primers for selected candidate genes and one primer pair for the reference gene, i.e., glyceraldehyde 3-phosphate dehydrogenase (GAPDH)^59^, are listed in Supplementary Table 7. The reaction efficiency of each pair of primers was determined by gradient dilution of the template. A five-gradient dilution of cDNA was used as a template to create the standard curves. Reaction efficiency was calculated from the slope of the standard curve using the formula: reaction efficiency = [10 (− 1/slope)] − 1 × 100. The reaction efficiency for each pair of primers was between 95 and 113% (Supplementary Table 7). For qRT-PCR, the leaf samples were collected from two genotypes (ZZ1 and GT46) at 1-month post-inoculation. ZZ1 is a resistant genotype identified in several studies, while the GT46 has been reported to be susceptible to leaf scald^7^. Samples were quickly packed into foil and frozen in liquid nitrogen within 10 s of collection. Total RNA was extracted using the TRIzol reagent (ThermoFisher), following the manufacturer’s instructions. The qRT-PCR assay was performed on a Lightcycler480 (Roche) Real-Time PCR Detection System using the ChamQ SYBR qPCR Master Mix (Vazyme, Q311-02). Each reaction contained 10 µL of the Master Mix, 0.4 µL each of the forward and reverse primers, 0.4 µL of ROX Reference Dye II, 1.0 µL of the cDNA sample, and distilled water to the final volume of 20 µL. The qRT-PCR conditions consisted of pre-denaturation at 95 °C for 5 min, followed by 40 cycles of 95 °C for 15 s, 60 °C for 25 s, and 72 °C for 25 s. At the end of the PCR cycles, melting curve analysis was performed to validate the specificity of the PCR product. Three independent biological replicates, each with three technical replicates, were analyzed respectively. The relative gene expression levels were calculated using the 2^−ΔΔCt^ method^60^. The Ct values of internal reference genes were used to standardize the Ct values of target genes for all samples. Multiple comparisons were performed using one-way analysis of variance (ANOVA) followed by Turkey multiple comparison test.

We confirm that the use of plants in the present study complies with international, national, and institutional guidelines.

### Supplementary Information

Supplementary Legends.

Supplementary Figure 1.

Supplementary Figure 2.

Supplementary Figure 3.

Supplementary Figure 4.

Supplementary Figure 5.

Supplementary Tables.

## Data Availability

Phenotypic data from the current study are included in the Supplementary Files, and genotypic data are available from the corresponding author upon reasonable request.
