# Supplementary material for: Identification of candidate single-nucleotide polymorphisms (SNPs) and genes associated with sugarcane leaf scald disease
Source: Sci Rep. 2024 Jul 13;14:16214. doi: 10.1038/s41598-024-67059-w (PMC11246479; doi:10.1038/s41598-024-67059-w)
Supplement: Supplementary file 1 — Supplementary Legends. [file 41598_2024_67059_MOESM1_ESM.docx]

***Supplementary_Material***

**Identification of candidate single-nucleotide polymorphisms (SNPs) and genes associated with sugarcane leaf scald disease**

**Yisha Li*, Pingping Lin, Qian You, Jiangfeng Huang, Wei Yao, Jianping Wang, Muqing Zhang***

*** Correspondence:** Prof. Muqing Zhang : mqzhang@ufl.edu or zmuqing@163.com

**Supplementary Figures and Tables**

Supplementary Fig. 1 The frequency distribution of the raw phenotypic data for leaf scald resistance of each experiment.

Supplementary Fig. 2 The curve of cross‐validation error (CV error) values under different K values.

Supplementary Fig. 3 A heatmap of kinship matrix presenting genetic relatedness of 170 genotypes.

Supplementary Fig. 4 Quantile–quantile (Q–Q) plots corresponding to GWAS for leaf scald resistance.

Supplementary Table 1. List of disease severity of leaf scald evaluated over three environments.

Supplementary Table 2. Analysis of variance and estimates of heritability for leaf scald in 170 sugarcane genotypes.

Supplementary Table 3. List of SNP markers significantly associated with leaf scald disease resistance.

Supplementary Table 4. Candidate genes homologous to *S. officinarum* and *S. spontaneum* gene.

Supplementary Table 5. Primers used for the quantitative reverse transcription-PCR (qRT-PCR) assay.
