# Supplementary figures and images for: Identification of candidate single-nucleotide polymorphisms (SNPs) and genes associated with sugarcane leaf scald disease
Source: Sci Rep. 2024 Jul 13;14:16214. doi: 10.1038/s41598-024-67059-w (PMC11246479; doi:10.1038/s41598-024-67059-w)

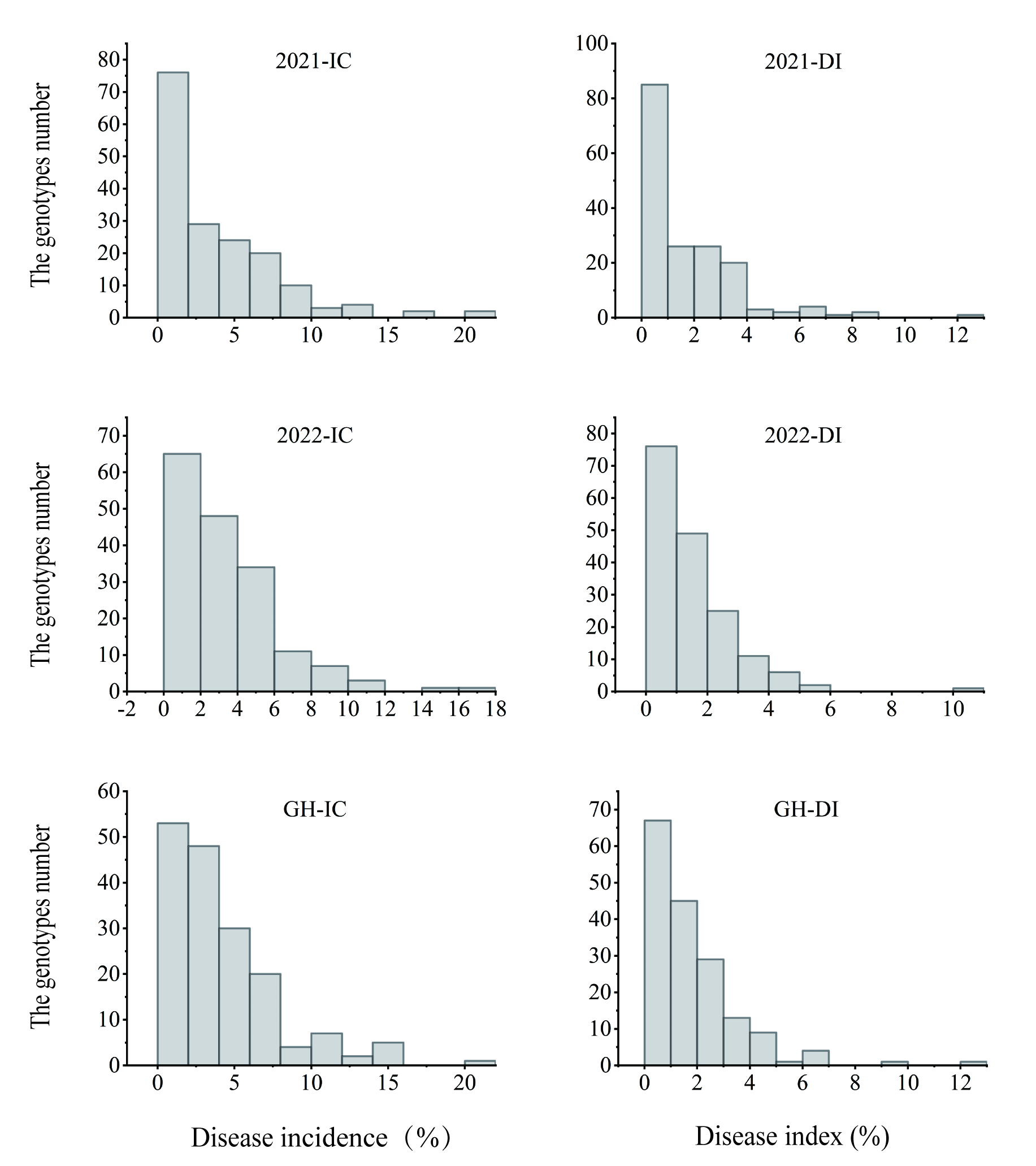

Supplement: Supplementary file 2 — Supplementary Figure 1. [file 41598_2024_67059_MOESM2_ESM.tif]

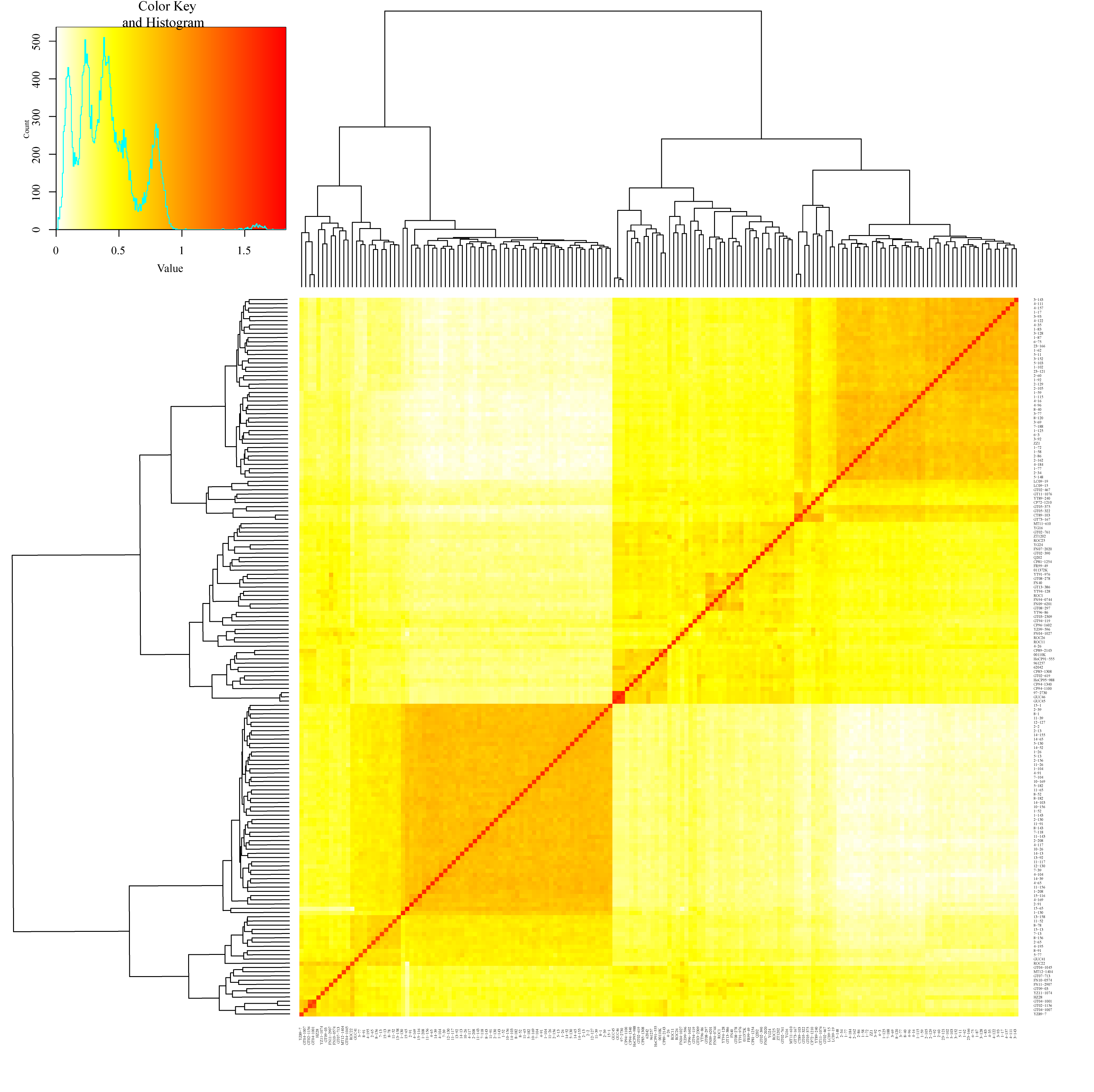

Supplement: Supplementary file 3 — Supplementary Figure 2. [file 41598_2024_67059_MOESM3_ESM.tif]

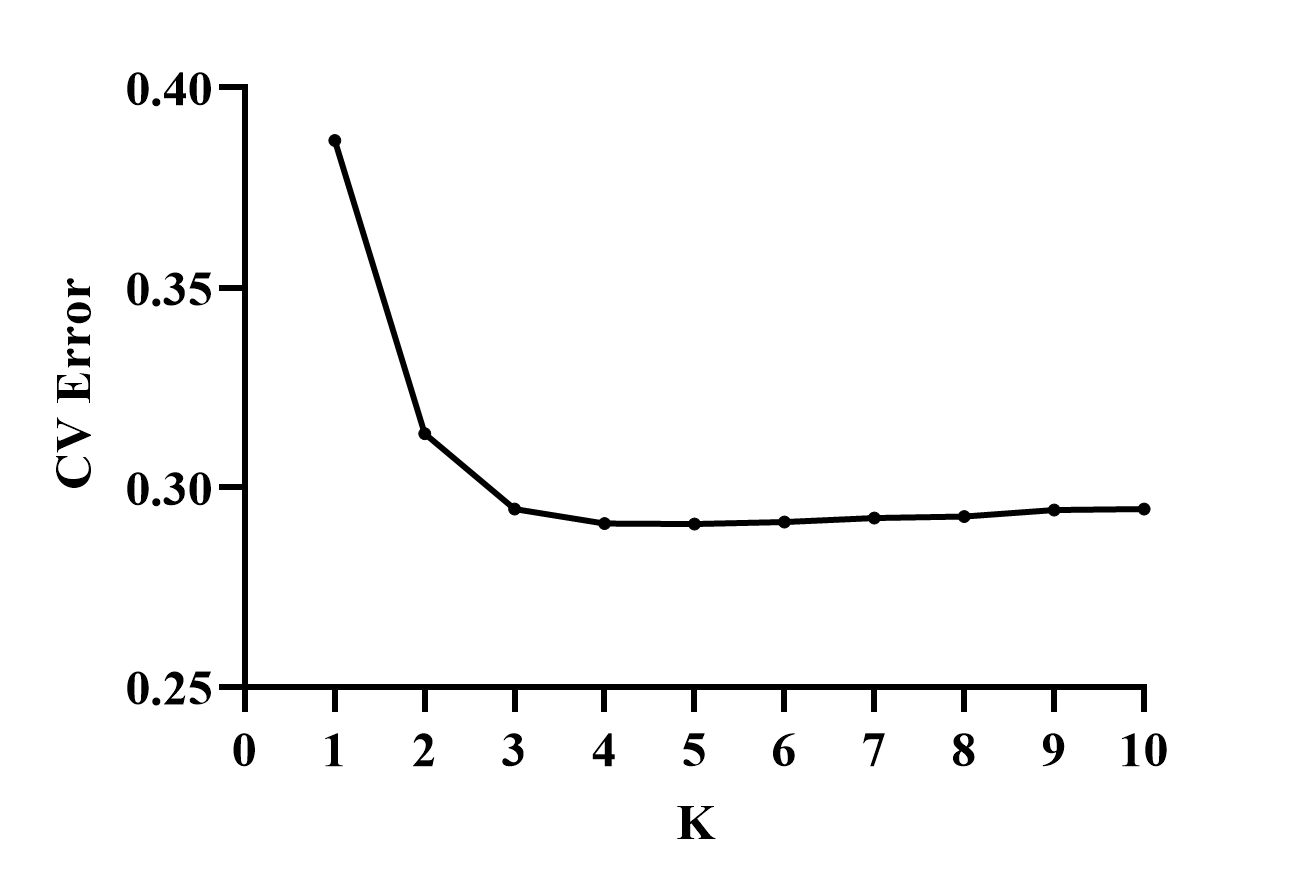

Supplement: Supplementary file 4 — Supplementary Figure 3. [file 41598_2024_67059_MOESM4_ESM.tif]

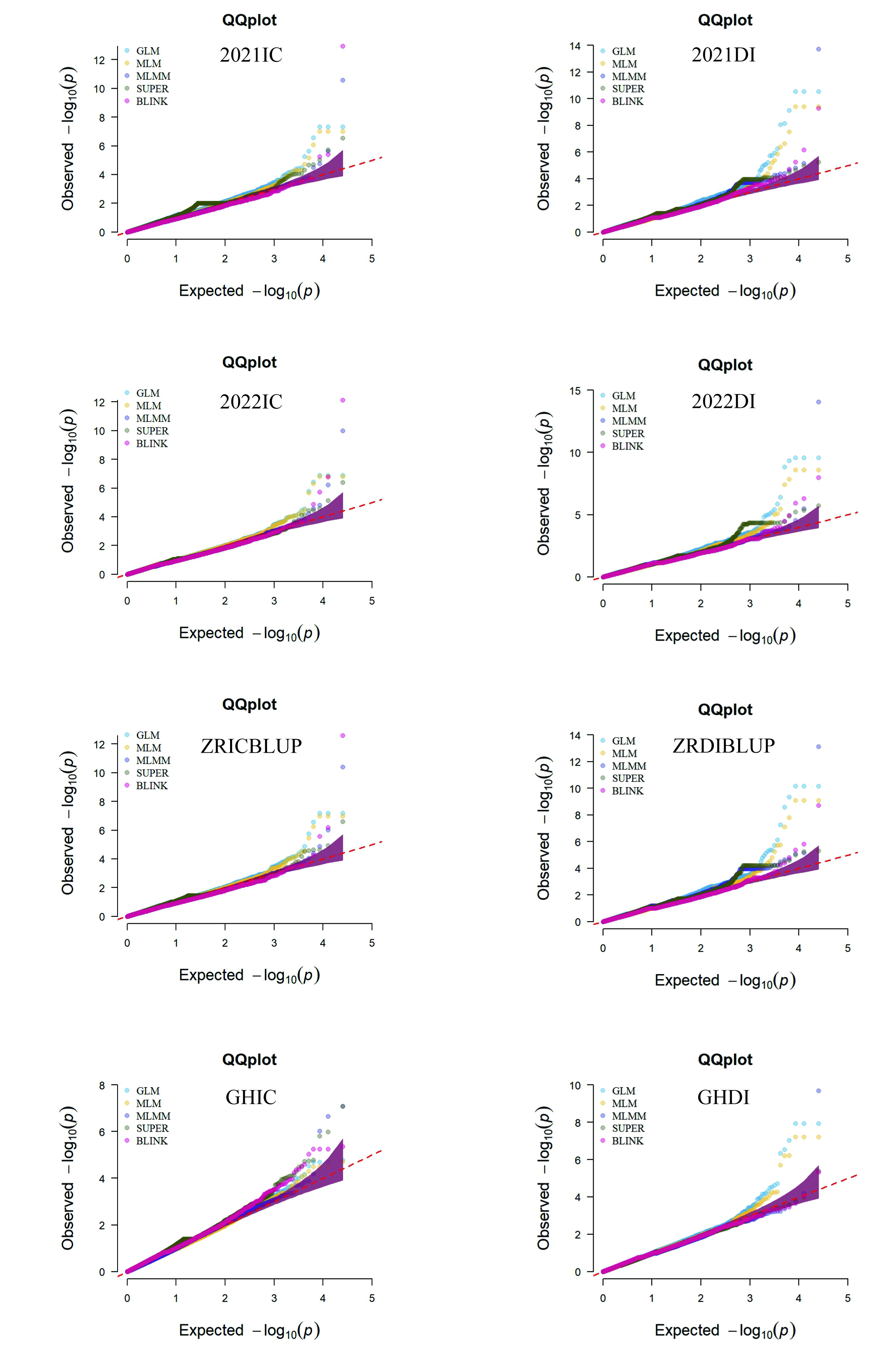

Supplement: Supplementary file 5 — Supplementary Figure 4. [file 41598_2024_67059_MOESM5_ESM.tif]

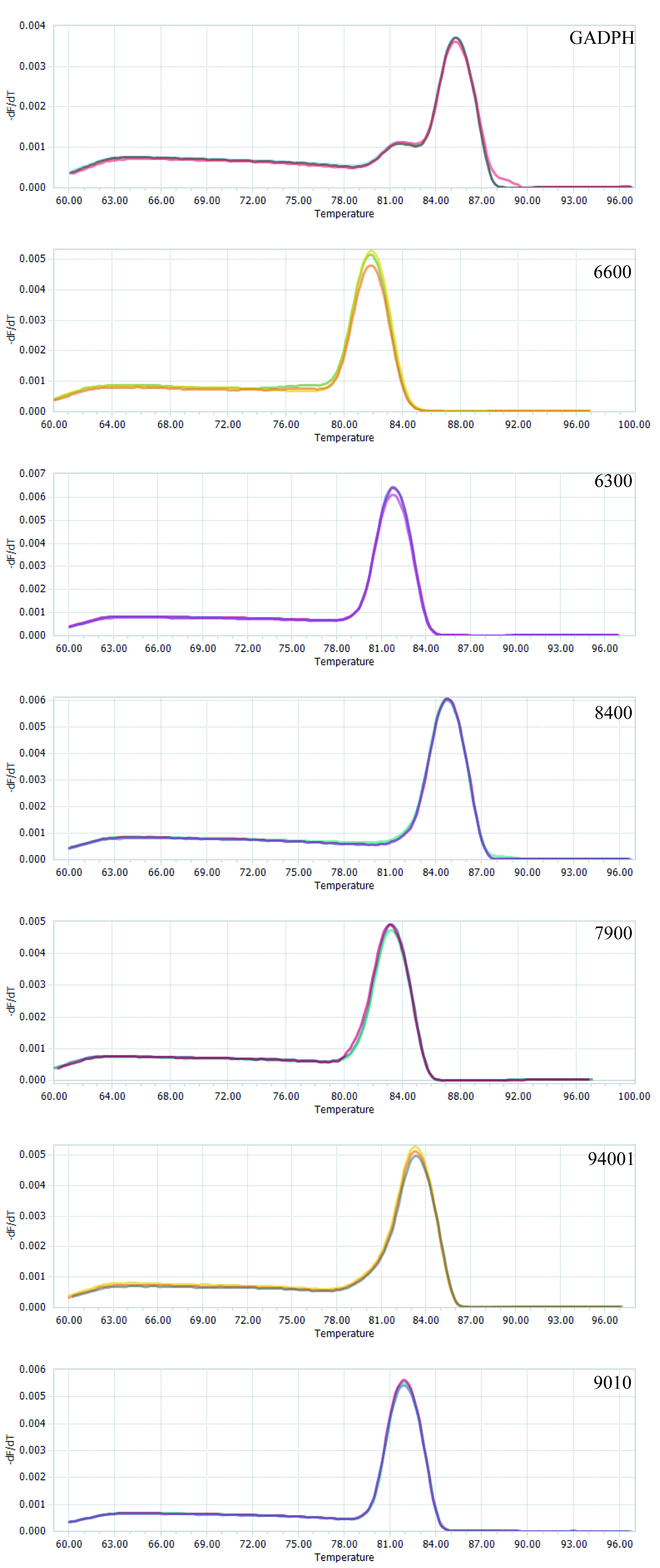

Supplement: Supplementary file 6 — Supplementary Figure 5. [file 41598_2024_67059_MOESM6_ESM.tif]
